# Deoxynivalenol and Zearalenone—Synergistic or Antagonistic Agri-Food Chain Co-Contaminants?

**DOI:** 10.3390/toxins13080561

**Published:** 2021-08-11

**Authors:** Asmita Thapa, Karina A. Horgan, Blánaid White, Dermot Walls

**Affiliations:** 1 School of Chemical Sciences, Dublin City University, Dublin 9, Ireland; asmita.thapa3@mail.dcu.ie; 2 Alltech Biotechnology Centre, Co. Meath, Ireland; khorgan@alltech.com; 3 School of Chemical Sciences, National Centre for Sensor Research, DCU Water Institute, Dublin City University, Dublin 9, Ireland; 4 School of Biotechnology, National Centre for Sensor Research, Dublin City University, Dublin 9, Ireland

**Keywords:** Deoxynivalenol, Zearalenone, synergistic, antagonistic, toxicity, co-occurrence

## Abstract

Deoxynivalenol (DON) and Zearalenone (ZEN) are two commonly co-occurring mycotoxins produced by members of the genus *Fusarium*. As important food chain contaminants, these can adversely affect both human and animal health. Critically, as they are formed prior to harvesting, their occurrence cannot be eliminated during food production, leading to ongoing contamination challenges. DON is one of the most commonly occurring mycotoxins and is found as a contaminant of cereal grains that are consumed by humans and animals. Consumption of DON-contaminated feed can result in vomiting, diarrhoea, refusal of feed, and reduced weight gain in animals. ZEN is an oestrogenic mycotoxin that has been shown to have a negative effect on the reproductive function of animals. Individually, their mode of action and impacts have been well-studied; however, their co-occurrence is less well understood. This common co-occurrence of DON and ZEN makes it a critical issue for the Agri-Food industry, with a fundamental understanding required to develop mitigation strategies. To address this issue, in this targeted review, we appraise what is known of the mechanisms of action of DON and ZEN with particular attention to studies that have assessed their toxic effects when present together. We demonstrate that parameters that impact toxicity include species and cell type, relative concentration, exposure time and administration methods, and we highlight additional research required to further elucidate mechanisms of action and mitigation strategies.

## 1. Introduction

Mycotoxins are structurally diverse, low-molecular-weight, fungal secondary metabolites that are harmful at low concentrations to farm animals and humans [1,2,3]. They are often found in many staple foods, including maize, cereals, and nuts. Mycotoxins easily enter the food chain following mould contamination of animal feed, the raw materials for these feeds, and human food sources [1,3,4]. Ingestion of mycotoxins can result in their accumulation in organs and tissues, which is a cause of concern due to their known toxicity and potential immunosuppressive and carcinogenic effects [5]. It has been estimated by the Food and Agriculture Organisation (FAO) of the United Nations that at least 25% of the world’s crops are contaminated with mycotoxins in any given year [6], although it is important to note that such data would vary from year to year and depend on the sensitivity of the methodology utilized.

Chemical, physical, and biological factors can affect the growth of moulds, and ultimately, mycotoxin production on contaminated crops. Physical conditions, including rainfall and temperature, along with chemical factors such as carbon dioxide, oxygen, and pesticide levels, can all affect mycotoxin production. Plant variety, plant stress, and insect attack can also affect mycotoxin levels [7]. Mycotoxin contamination can occur in the field, during harvesting, and in particular, during storage, if the right conditions for fungal growth are met [7,8,9]. The major mycotoxins and the fungal species that produce them, as well as the effects that they have on human health are shown in Table 1. Deoxynivalenol (DON) and Zearalenone (ZEN) are two of the most important mycotoxins of relevance to the agri-food industry and the human food chain, and considerable research efforts have been expended on deciphering their modes of action. Although co-occurrence of these mycotoxins is known to be universal, as illustrated in this review of published research studies investigating their impact, there is considerable evidence to show that the combined effects of these mycotoxins cannot be predicted from the effects observed when they are studied individually.

## 2. Deoxynivalenol

**Figure 1:**
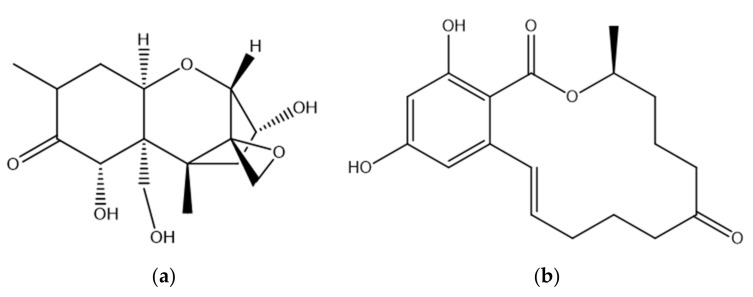
Structure of Deoxynivalenol (**a**) and Zearalenone (**b**) [16,24].

DON is a mycotoxin produced in crop grains infected with *F. graminearum* and *F. culmorum.* It is found mostly in wheat, barley, rye, and corn. DON is the most commonly found type B trichothecene, and its structure is shown in Figure 1 [15]. DON has two secondary and one primary hydroxyl groups present along with an epoxide and a conjugated ketone, either of which may be associated with toxicity [16]. The epoxide on the C12/13 position of DON is considered to be essential for toxicity and has a key role in the inhibition of cellular protein synthesis [17,18,19]. It has been shown that the opening of the epoxide results in a loss of DON toxicity [20]. Other studies that point to a role for this group have shown that the de-epoxy metabolites of DON are less toxic than DON itself [21,22,23]. In one study, the cytotoxicity of DON and another trichothecene, Nivalenol, and its de-epoxy metabolite were compared using a Bromodeoxyuridine based cell proliferation assay. The results illustrated that the de-epoxy metabolites of DON and Nivalenol were 54 and 55 times less toxic, respectively, than the toxins with the epoxide ring [21]. Similar results were obtained in another study in which the cytotoxicity of DON and deepoxy-deoxynivalenol were compared, and in which the relative lack of deepoxy-deoxynivalenol toxicity was confirmed using six different cytotoxicity assays [23]. These studies clearly show that the 12, 13-epoxide ring plays an important role in DON toxicity.

The primary mode of action of DON and other trichothecenes is the inhibition of protein synthesis. Both an intact C12-13 epoxide and a double bond at C9-10 position are essential for this inhibitory activity. DON binds to the 60 S subunit of the eukaryotic ribosome and interferes with the action of peptidyl transferase [25,26]. In one study using human intestinal epithelial Caco-2 cells (derived from colorectal carcinoma tissue), it was found that there was a concentration-dependent effect of DON on total cellular protein synthesis and content [27]. Another study investigated the effect of DON on protein synthesis in pig tissues. Pigs were fed a DON contaminated diet where the DON concentrations used were 2 µg/kg (control group), 77 µg/kg (chronic oral), 83 µg/kg (acute oral) and 53 µg/kg (acute intravenous). The results showed a significant reduction in overall protein synthesis in the kidneys, spleen and ileum of DON exposed pigs [28]. In another study, the mRNA levels of IFN-γ, IL-1β and TGFBR1 were down regulated by DON in jejunal tissues of broiler chickens [29]. In both humans and animals therefore, the inhibition of protein synthesis has been an observed mode of action.

### 2.1. DON Occurrence

Although DON is not the most toxic member of the trichothecenes, it is one of the most commonly found and studies have shown that it can be globally present in wheat, barley, maize and rice amongst others [30,31]. In a three-year study conducted between 2009 and 2011, DON was founded to be present in over half (59%) of 7049 samples analysed from American, European and Asian sources including soybean, wheat, and dried distillers grains with soluble and finished feed [32]. Toxin occurrence data should always be treated with some caution due to the difficulty in ensuring that samples taken for analysis are representative. Furthermore, misestimation can occur due to the presence of masked or bound toxins present in samples’. The frequent occurrence of DON in food and animal feed however is clearly a problem in both the food and livestock industries [33]. An additional cause of concern is that DON is a very stable compound, both during storage and food preparation stages [34]. The European Food Safety Authority (EFSA) has set guidelines for the maximum levels of DON allowable in various foodstuffs (see Table 2). The allowable limits for DON in food and feed outside of Europe can vary. In Indonesia, the maximum limit for DON in maize and wheat is 1000 µg/kg, for pasta and noodles is 750 µg/kg, and 500 µg/kg for ready to eat products such as pastry, bread and biscuits. In Japan the limit for DON in wheat is 1100 µg/kg. In South Korea, the limit for grain and their processed foods, corn and their processed foods and cereals is 1100 µg/kg, 2000 µg/kg and 500 µg/kg, respectively [35]. In the US, the limit for DON in finished wheat products is 1000 µg/kg and in grains and grain by-products for swine is 5000 µg/kg, which is considerably higher than that for Europe (900 µg/kg) [36].

It has been found that low temperatures along with high humidity and heavy rainfall can lead to increased contamination levels of DON [39,40]. A study by Hoogenboom et al. involving wheat samples collected in 2003 and 2004 from the Netherlands revealed that levels of DON found in the 2003 samples was less than 1000 µg/kg, below the EFSA maximum level [41]. However, for the samples collected in August 2004, after a period of heavy rainfall, DON levels as high as 11,000 µg/kg, by far in exceedance of maximum EC levels, were found [41]. In Luxemburg (2007 and 2008), 75% of 33 fields of winter wheat sampled were found to have DON contamination. Nine percent of these exceeded the recommended EFSA maximum level of DON for unprocessed wheat [42]. In a study in The Netherlands, 57 out of 86 samples that were collected from winter wheat fields in 2009 tested positive for DON with 2524 µg/kg recorded in one case [43]. In Argentina, 85% of 120 samples of freshly harvested wheat from nine locations in the Northern Buenos Aires Province (2004) were contaminated with DON with one sample reaching 2788 µg/kg [44]. In the Jiangsu province of China 74.4% of 180 wheat samples harvested in 2010–2012 tested positive for DON [45]. Another study reported that 16 out of 23 South African wheat flour samples taken during the latter half of 2006 were contaminated with DON [46]. These findings highlight the global occurrence of DON as a food chain contaminant and underscore the importance of efforts to both prevent contamination and mitigate against the effects of this toxin [31].

### 2.2. DON Toxicity

DON is toxic to both humans and animals when ingested. It mainly affects the gastrointestinal tract and immune system resulting in nausea, diarrhoea and vomiting [27,30]. Amongst farmed animals, pigs are particularly sensitive to DON with one study linking the consumption of DON contaminated feed (at 2800 µg/kg) to decreased feed intake and a reduction in weight gain [47]. Pig jejunal cells exposed to DON have shown time and dose dependent toxicity responses, based observations of cell morphology, following treatments in which up to 5 µM DON was used for up to 8 h [48]. Elsewhere, pig jejunal cells also showed reduced total cell counts and increased lactate dehydrogenase release following 48 h DON exposure over a concentration range of 0–10 µM [49]. There are also reports of genotoxic effects associated with DON exposure. The comet assay was used to investigate DNA damage due to DON in human liver (HepG2) carcinoma cells. The results showed that DNA damage was induced in a dose dependent manner following exposure to DON in as little as 1 h [50]. These findings are consistent with another report in which Caco-2 cells exposed to 0.01–0.5 µM DON for 24 to 72 h also showed evidence of DNA damage by comet assay. The average tail moment, which represents the extent of DNA damage, for untreated Caco-2 cells was 1.23 ± 0.73, a value that increased to 4.11 ± 1.53 when the cells were incubated with 0.1 µM DON for 24 h. When the cells were exposed to 0 and 0.1 µM DON for 72 h, the average tail moment was 1.09 ± 0.31 and 4.6 ± 0.81 respectively [51]. In contrast, DON-associated genotoxicity was investigated in vivo in seven mouse organs (duodenum, colon, blood, liver, spleen, kidney, bone marrow), following a 3-day oral administration of DON. The results of the comet assay failed to show evidence of DNA damage at up to 53.9 µM DON. The same study also used human lymphoblast TK6 cells exposed to DON at concentrations of up to 25 µM for 3 and 24 h. Again, the results showed that there was no increase in DNA damage at both exposure times [52].

The intestine is a major site of DON absorption as it is exposed to contaminated feed. The intestinal epithelium acts as a frontier barrier to the external environment including harmful toxins [53,54]. DON has been shown to alter the intestinal barrier function by affecting the trans-epithelial electrical resistance (TEER) which is a reliable indicator of barrier integrity and permeability [54,55]. A significant time and dose dependent reduction of the TEER value of Caco-2 cells was observed following treatment with 5 µM DON (a decrease of 19% after a 14 day exposure) and higher DON concentrations had more drastic negative effects on TEER values [56]. A similar trend was also observed using two porcine intestinal epithelial cells, IPEC-1 [56] and IPEC-J2 [57]. For IPEC-1 cells, there was a 25% and 60% decrease in the TEER value when the cells were exposed to 10 and 50 µM DON respectively over 24 h. After exposure for 14 days, the TEER value decreased by 58% and 97% for 10 and 50 µM respectively [56]. For IPEC-J2 cells, there was also a significant decrease in the TEER value with 20 µM DON at 4, 8, 12 and 24 h [57]. In summary, there are published reports, which show that DON is cytotoxic to different cell types and tissues with some evidence of cell type dependent genotoxicity and that it is effective in disrupting the integrity of the intestinal epithelium.

## 3. Zearalenone

ZEN is one of the most important *Fusarium* mycotoxins, produced by several species including *F. graminearum, F. culmorum, F. cerealis* and *F. equiseti* [58,59]. It is a non-steroidal oestrogenic mycotoxin found mainly in corn, wheat, oats, barley and sesame seeds [58,60]. The production of ZEN is greatest at cool temperatures and high humidity [61]. It is a stable compound that does not degrade during storage and food preparation or at high temperatures [62]. ZEN has a resorcyclic acid lactone structure and is similar to that of naturally occurring oestrogens such as 17-β-oestradiol (Figure 1). ZEN is classified as a xenoestrogen as it mimics the activity of oestrogens by binding to mammalian oestrogen receptors (ERs) [63]. This ER engagement leads to disruption of endocrine function which can lead to disorders of the reproductive system [64]. Consumption of feed contaminated with ZEN therefore alters the normal hormonal balance leading to a perturbance of the reproductive system in farm animals [13,65]. ZEN can bind to ERs in the cellular cytoplasm resulting in lipid peroxidation with concomitant cytotoxic effects. ZEN can also engage ERs on the surface of immune cells which in turn can interfere with immune responses [66]. ZEN has been shown to be hepatotoxic, genotoxic and to cause immunosuppression [24]. It has been classified as a group 3 carcinogen (‘not classifiable as to its carcinogenicity to humans’) by the International Agency for Research on Cancer [67]. Following oral exposure, ZEN is absorbed rapidly and is metabolised in the liver. There are two major pathways for the biotransformation of ZEN in animals [68]. Biotransformation via hydroxylation results in the formation of the two major metabolites of ZEN, namely α-zearalenol (α-ZOL) and β- zearalenol (β-ZOL), which are believed to be catalysed by 3α- and 3β-hydroxysteroid dehydrogenases. The second pathway is the conjugation of ZEN and its reduced metabolites with glucuronic acid and sulphate resulting in the formation of the metabolites ZEA-14-O-glucoside, ZEA-16-O-glucoside and ZEA-14-sulphate [13,24,60,65,68]. Microbial metabolism of toxins has been shown to take place in the intestine of ruminant animals. In the case of ZEN, such ruminal metabolism may lead to higher circulatory concentrations of α-ZOL following absorption and prior to liver metabolism occurring [69,70,71,72,73]. α-ZOL and β-ZOL are also produced by *Fusarium*, but at much lower concentrations than ZEN and they differ in their binding affinities to ERs [74]. α-ZOL is known to have a higher affinity for ERs than ZEN and its other metabolites [75]. ZEN is more reactive and toxic in species where the biotransformation to α-ZOL is preferred [76]. The metabolism of ZEN is highly dependent on the animal species and by differences in the quantities of oestrogen receptors present. Pigs are more susceptible to ZEN toxicity than any other domestic animals due to the fact that the toxin is mainly metabolised to α-ZOL. In contrast, ZEN is mostly converted to β-ZOL in cattle thus making them less susceptible to its toxicity [74,77].

### 3.1. ZEN Occurrence

ZEN is commonly found contaminating cereals in warm and temperate climates. In the Republic of Serbia, ZEN was detected in 12%, 37%, 100% and 53% of maize samples in 2012, 2013, 2014 and 2015, respectively. The weather conditions for each year were also analysed in the same study and it was found that the detection of ZEN in all of the 2014 samples correlated with extreme wet weather conditions in that year [78]. Another study also showed that the presence of ZEN in Romanian wheat was elevated with higher amounts of rainfall [79]. In a ten-year study (2008–2017), samples of feed and feed raw materials were collected from 100 countries and analysed for the presence of mycotoxins. The data showed that ZEN was detectable in 45% of the total of 61,413 samples tested. The study revealed variations in ZEN concentrations and a potential correlation with the amount of rainfall that occurred in a given year [80]. The occurrence of ZEN in various food products has been noted in several reports. In a Turkish study, ZEN was found in 4% of 50 wheat samples, 20% of 15 maize samples, 55% of 20 paddy rice samples and 4% of 50 wheat flour samples [81]. An analysis of corn meal that was produced in Brazil, found that of the 84 samples that were examined, 78.6% (66) tested positive for ZEN [82]. In India, an analysis of samples of corn, rice, wheat and oats from local markets found that 84% of the 117 samples were contaminated with ZEN, with 33% of samples exceeding the EU permissible limit [83]. In another study, 43% of maize kernel samples from Poland (2011 and 2012) tested positive for ZEN [84]. Due to the global occurrence of ZEN, the EFSA has set guidelines for the maximum levels of ZEN allowable in various foodstuffs (Table 3). The limit for ZEN in food and feed can vary outside of Europe. In Japan, this value is set at 1000 µg/kg ZEN for compound feeds. In South Korea, the limit for ZEN in grains and processed grain foods, confectionaries and baby foods is 200 µg/kg, 50 µg/kg and 20 µg/kg, respectively [35].

### 3.2. ZEN Toxicity

ZEN toxicity has been observed in a range of cell types. ZEN was shown to inhibit IPEC-J2 cell proliferation at 40 µM and to decrease cell viability at all concentrations used (up to 100 µM) in a 24 h period [85]. A concentration dependent decrease in cell viability was also observed when Caco-2 cells were insulted with ZEN (1–150 µM) over a 72 h period [86]. These results agreed with another study in which liver-derived HepG2 cells showed a dose-dependent decrease in viability in response to ZEN exposure after 24 h (48% and 11% at 200 and 100 µM ZEN, respectively) [87]. HepG2 cells were also used in a more recent study where the cytotoxicity of ZEN and its metabolites α-ZOL and β- ZOL was assessed using the neutral red assay. The results showed a dose-dependent decrease in cell viability in all cases with β- ZOL being the most toxic based on the IC_50_ values, followed by ZEN and α-ZOL [88]. Other studies have shown that lower doses of ZEN can promote increased cell proliferation whereas higher doses can result in decreased cell viability and death. After 120 h of exposure, ZEN enhanced the proliferation of human colon carcinoma cells (HCT116) when used at 1 nM to 1 µM. At 48 h however, the methylene blue assay showed a dose-dependent inhibition of cell viability when concentrations higher than 20 µM were used [89]. A similar trend was observed in another study using IPEC-J2 cells whereby increased cell viability was observed after 48h with 10 µM ZEN, whereas decreased viability was observed over the same time period when 40 µM ZEN was used [90].

ZEN-associated genotoxicity has also been reported. In one study using HEK293 cells (derived from human embryonic kidney), the comet assay was used to show a dose-dependent increase in DNA damage at 2 h in response to ZEN (up to 20 µM) [91]. Another study reported the induction of DNA damage by ZEN in SH-SY5Y human neuroblastoma cells [92]. Elsewhere, the comet assay in conjunction with bacterial DNA repair endonucleases was used to show ZEN induced oxidative DNA damage in HepG2 cells [93]. ZEN has been shown to induce programmed cell death (apoptosis) in bovine mammary epithelial cells [94] and porcine granulosa cells [95]. Apoptosis was also observed when rat sertoli cells were treated with ZEN. In that study, insult with ZEN coincided with upregulation of the pro-apoptotic proteins Bid and Bax in conjunction with down-regulation of the anti-apoptotic Bcl-2, [96]. Another report described the effect of ZEN on the intestine of juvenile grass carp. It was found that fish diets spiked with ZEN concentrations greater than or equal to 1548 µg/kg led to a pro-apoptotic shift in the balance of Bcl-2 family members followed by the expression of caspases and the onset of apoptosis in fish intestines [97]. In summary, it can be concluded that ZEN has time and dose-dependent negative effects on a range of cell types.

## 4. Co-Occurrence of DON and ZEN

DON and ZEN are both produced by *Fusarium culmorum* and it is not surprising therefore that they are frequently to be found in co-occurrence with each other [98]. In Portugal DON and ZEN were found to co-occur in 15% (46/307) of wheat and wheat-based products with a mean ZEN concentration of 170 µg/kg and mean DON concentration of 70 µg/kg [99]. In another study, DON and ZEN were seen to co-occur in Brazilian barley grain samples [100]. Co-occurrence was also reported in wheat from Brazil. There, a combination of DON, ZEN and nivalenol was found in 74% (2009) and 12% (2010) of wheat samples analysed [101]. A global mycotoxin survey revealed that the combinations of DON plus ZEN, DON plus fumonisins (other Fusarium produced mycotoxins) and ZEN plus fumonisins had the highest percentage of co-occurrence in finished feed (48%, 48% and 43% respectively) and that DON and ZEN were found to co-occur in 39% and 28% of maize and wheat samples analysed, respectively [80]. Elsewhere, DON and ZEN were shown to have a co-occurrence of 35% in fish feed samples taken in Kenya [102]. One study analysed the co-occurrence DON and ZEN in feeds in China during 2018–2020. The results showed that DON and ZEN co-contaminated 100% of rapeseed meal, peanut meal, grass grain, fish meal, wheat flour, rice bran, corn bran, corn gluten meal, corn germ meal and soybean bran in each of the years tested. DON and ZEN were shown to co-occur in 97.8, 96.7 and 100% of complete pig feed samples taken in 2018, 2019 and 2020, respectively. In poultry feed, these mycotoxins were also found to co-occur in 98.8%, 100% and 100% of the samples tested in 2018, 2019 and 2020, respectively [103].

## 5. In Vitro Studies on Co-Exposure to DON and ZEN

The combined toxic effects of DON and ZEN have been assessed in vitro in various reports in which a range of cell types were used. The specific details of these are summarised in Table 4. Liver cells have received much attention due to the important role of this organ in toxin metabolism. The detoxification of DON occurs in the liver with the formation of DON-glucuronide in both humans and animals [104]. The biotransformation of ZEN to its major metabolites α-ZOL and β- ZOL also occurs in the liver [13]. Using HepaRG human hepatic cells, lower DON and ZEN co-exposure times (3 h and 12 h) resulted in significant cell mortality, which was not observed when each mycotoxin was used individually, thus indicating a synergistic effect. At 18 h, ZEN alone was found to induce apoptosis and necrosis whereas there was no significant cell mortality observed due to DON alone. Once the cells were exposed to both mycotoxins, the effect was similar to that of ZEN alone indicating an additive effect [105]. In another study by the same group, DON was shown to be cytotoxic to HepaRG after 14 days exposure whereas ZEN did not have a significant effect on cell viability even after exposure for 42 days. When the mycotoxins were combined, the effect was found to be the same as for exposure to DON alone and hence the effect of co-exposure was considered to be additive [106]. In another study with HepaRG, the cell proteome was analysed at 1 h and 24 h following mycotoxin exposure. Although significant changes in the proteome were observed in response to DON, ZEN and DON plus ZEN, these were not consistent between mycotoxin exposures. After 1 h, the effect of co-exposure appeared to be synergistic whereas an antagonistic effect was observed at the later exposure time of 24 h. It was also concluded that the cellular response to ZEN induced stress at 24 h was reduced when it was combined with DON and that the observed antagonistic effect following the longer exposure time may have been due to possible mitigation by hepatocytes [107].

One study used a cell based electrochemical sensor to assess the effects of DON, ZEN and co-exposure to both at 24 h on human hepatocellular carcinoma (BEL-7402) cells. The data showed an additive effect upon co-exposure [108]. In a similar approach taken using HepG2 cells co-exposure at higher mycotoxin levels was seen to have a synergistic effect [109]. HepG2 cells were also used in another study that observed synergistic effects on cell viability at 48 h following co-exposure to DON and ZEN [110]. Liver toxicity due to DON and ZEN has also been studied in vivo. In one report using mice, the antioxidant capacity and the concentration of malondialdehyde was measured as a readout for oxidative stress induced by the DON and ZEN. The results showed that at two weeks following toxin administration, co-exposure had an antagonistic effect on both parameters relative to that observed following exposure to each toxin individually. Thus, it appeared that each mycotoxin induced more oxidative stress when administered individually than that observed when both were used together. In contrast however, it was found that ZEN plus DON exhibited a synergistic effect on pro-apoptotic *bax* mRNA levels and Caspase-3 enzyme activity in the liver [111]. In summary, these studies have shown that toxicity following co-exposure of liver cells to DON and ZEN cannot easily be predicted from the effects observed when both toxins are used individually.

**Table 4:** Combinatorial interaction between DON and ZEN.

**Cell Line Used**	**Toxin Levels**	**Exposure Time and Route**	**Conclusions**	**Observations**	**Comment**	**References**
HepaRG	DON 0.2–10 µM ZEN 1.5–75 µM	48 h	Synergistic at 48 h cell viability	Cell viability	Doses correspond to IC50 values after 48 h	[105]
	DON 7.35 µM ZEN 55.1 µM	18 h	Additive at 18 h cell mortality	Cell mortality		
HepaRG	DON 2.5 µM ZEN 0.24 µM	14 days 28 days 42 days	Additive	Cell viability	Doses correspond to maximum levels permitted in cereals for humans	[106]
HepaRG	DON 0.2 µM ZEN 20 µM	1 and 24 h	Synergistic at 1 h Antagonistic at 24 h	Cell proteome	Doses correspond to IC10 values after 48 h	[107]
BEL-7402	DON 0.37–16.9 µM ZEN 0.31–31.4 µM	24 h	Additive	Cell viability	Mixtures used DON + ZEN 0.37 + 0.31 µM 0.68 + 0.63 µM 1.69 + 1.57 µM 3.7 + 3.1 µM 6.8 + 15.7 µM 16.9 + 31.4 µM	[108]
HepG2	DON 0.34–67.5 µM ZEN 15.7–157 µM	24 h	Synergistic	Cell viability	Mixtures used DON + ZEN 0.034 + 15.7 µM 1.69 + 47.1 µM 3.7 + 62.8 µM 37 + 126 µM 67.5 + 157 µM	[109]
HepG2	DON 0.02–2 µM ZEN 0.28–34.5 µM	48 h	Synergistic	Cell viability	DON + ZEN 0.02 + 0.28 µM 0.03 + 0.53 µM 0.07 + 1.1 µM 0.14 + 3 µM 0.27 + 4.4 µM 0.51 + 8.6 µM 1 + 17.3 µM 2 + 34.5 µM	[110]
RAW 246.7	DON 0.0027–0.34 µM ZEN 0.28–37.69 µM	48 h	Synergistic	Cell viability	DON + ZEN 0.0027 + 0.28 µM 0.0054 + 0.6 µM 0.01 + 1.19 µM 0.02 + 2.36 µM 0.04 + 4.71 µM 0.08 + 9.42 µM 0.17 + 18.8 µM 0.34 + 37.69 µM	[110]
Caco-2	DON 3.3–16.7 µM ZEN 10–50 µM	24 h	Antagonistic	Cell viability	DON and ZEN combination in 1:3 ratio	[112]
HCT116	DON 100 µM ZEN 40 µM	24 h	Antagonistic	Cytotoxicity, mitochondrial apoptosis	Doses correspond to IC30 values after 24 h	[113]
		48 h		Cell cycle analysis		
IPEC-J2	Cytotoxic concentration DON 2 µM DON ZEN 40 µM	48 h	Cytotoxic concentration Reported as non-additive	Cell viability	Dose correspond to cytotoxic and non-cytotoxic concentrations	[90]
	Non-cytotoxic concentration DON 0.5 µM ZEN 10 µM		Non-cytotoxic concentration Synergistic			
PK15	DON 0.25 µM ZEN 20 µM	24 h	Synergistic	ROS levels Apoptosis	Doses used are concentrations close to IC10 concentration which were 0.157 and 27.583 µM for DON and ZEN, respectively	[114]
Porcine splenic lymphocytes	DON + ZEN 0.2 + 0.25 µM 1 + 1.26 µM 5.1 + 6.28 µM	48 h	Synergistic	Apoptosis Oxidative injury		[115]
Porcine lymphocytes	DON + ZEN 0.24 + 15.7 μM 0.71 + 31.4 μM	24/48/72 h	Antagonistic	Cell viability	Doses used were below IC50 concentration after 24, 48, 72 h exposure	[116]
			Antagonistic at lower concentration Synergistic at 72 h at higher concentration	Genotoxicity		
THP-1	DON 0.1–10 μM ZEN 2–100 μM	48 h	Antagonistic	Cell viability	DON + ZEN + 2 μM 0.8 + 16 μM 2 + 40 μM 4 + 80 μM 10 + 100 μM	[117]
ANA-1	DON 0–33.7 µM ZEN 0–37.7 µM	24 h	Synergistic	Cell viability and apoptosis	DON + ZEN concentration used for apoptosis and metabolism study 0.34 + 25.1 µM	[118]
			Antagonistic	Cell metabolism		
BF-2	DON 0–16.2 µM ZEN 0–120.3 µM	48 h	Antagonism	Cell viability fish Oxidative stress fish	DON + ZEN 0.13 + 1.33 0.25 + 2.66 0.51 + 5.32 1.01 + 10.64 2.02 + 21.29 4.05 + 42.58 8.1 + 85.15 16.2 + 170.3	[119]
Caco-2, HepaRG and THP-1 Co-culture	DON + ZEN Concentration used when Caco-2 cells were in luminal compartment: 1.6 + 24 µM 3 + 31 µM Concentration used when HepaRG cells were in luminal compartment: +20 µM 2.3 + 33 µM	48 h	No cytotoxicity with low concentration and in tri-culture Synergistic effect with higher concentration in bi-culture system	Cell viability		[120]
**Animal used**	**Toxin levels**	**Exposure time and route**	**Conclusions**	**Observations**	**Comment**	**References**
Zebrafish larvae	DON 67.5 µM ZEN 6.28 µM	72 h	Antagonistic	Cell mortality		[119]
Mice	DON 1500, 2500 µg/kg bodyweight ZEN 20,000, 30,000 µg/kg bodyweight	12 days Intraperitoneal injection	Antagonistic	Oxidative stress Renal apoptosis	DON + ZEN 1500 + 20,000 1500 + 30,000 2500 + 20,000 25 + 30,000 µg/kg bodyweight	[121]
Mice	DON 5000 µg/kg bodyweight ZEN 5000 µg/kg bodyweight	2 weeks Oral administration	Antagonistic	Oxidative stress	No change observed on liver weight	[111]
			Synergistic	Apoptosis		
Rats	DON 30 µg/animal/day ZEN 15 µg/animal/day	14 days Administered as a gavage dose	Antagonistic	Liver weight Glutathione level in liver Malondialdehyde level in kidney	Doses are according to EU limits in finished feed for young pigs	[122]
Mice	DON 0.5–2 μM ZEN 10–40 μM	24 h	Synergistic	Cell viability Immune function	DON and ZEN combined 1:20	[123]
Rats	DON 16.5 µg/animal/day ZEN 12.75 µg/animal/day	5 days Intraperitoneal administration	Synergistic	Glutathione and glutathione peroxidase activity in the liver	Doses correspond to 1 mg/kg diet for DON and 1.5 mg/kg diet for ZEN which are close to EU limits in finished feed for young pigs	[124]
Mice	DON 2000 mg/kg ZEN 20,000 mg/kg	21 days Intragastric administration	Antagonistic	Metabolic profiling of liver and serum		[125]
Mice	DON 2000 mg/kg ZEN 20,000 mg/kg	3 weeks Intragastric administration	Antagonistic	Metabolic pathway		[126]
Mice	DON 1500, 2500 µg/kg body weight ZEN 20,000, 30,000 µg/kg body weight	4 days Intraperitoneal injection	Synergistic	Apoptosis Antioxidant levels	DON + ZEN 1500 + 20,000 1500 + 30,000 2500 + 20,000 2500 + 30,000	[127]
Female piglets	DON 1000.6 µg/kg ZEN 269.1 µg/kg DON + ZEN 1007.5 + 265.4 µg/kg	3 weeks *Ad libitum* feeding	Synergistic	Body weight gain Average daily feed intake Intestinal functions	Barley naturally contaminated with DON and corn naturally contaminated with ZEN was used to manufacture the feed	[128]

The intestinal epithelium is the first barrier exposed to mycotoxins following the consumption of contaminated food or feed and so it is appropriate therefore that the toxicity of mycotoxins on various types of intestinal epithelial cell has been studied [129,130,131]. The CCK-2 assay was used to measure the viability of Caco-2 cells following challenge with DON and ZEN. Here, both mycotoxins behaved antagonistically in that the level of cytotoxicity observed during co-exposure was lower than that seen when each was used individually [112]. Elsewhere, an antagonistic effect during co-exposure to DON and ZEN was also observed with HCT116 cells following an analysis of cytotoxicity, apoptosis induction and cell cycle analysis [113]. Another study exposed IPEC-J2 cells to DON and ZEN both individually and combined at non-cytotoxic concentrations of each for 48 h. However when both toxins were combined at these non-cytotoxic concentrations, the authors observed a cytotoxic response and thus concluded a synergistic effect [90]. At cytotoxic concentrations of each toxin however, the effect of co-exposure was seen to be non-additive and it was concluded that in mixtures containing DON, there were no increases in overall cytotoxicity but in mixtures containing ZEN all of the mixtures were more cytotoxic than when ZEN alone was used. Interpretations of these results by others have varied, including that DON and ZEN were having a synergistic effect [111,119,127], the toxins were synergistic at cytotoxic concentrations [105,123], or were synergistic at cytotoxic concentrations and antagonistic at non-cytotoxic concentrations [132], were antagonistic [128] and antagonistic at the lower dose [116]. In addition to damage to liver and intestinal cells, some mycotoxins have also been shown to induce nephrotoxicity. A study using PK15 cells, derived from porcine kidney, showed a higher level of ROS production when the cells were co-exposed to DON and ZEN than when treated with each individual toxin. Similarly, co-exposure also led to a greater apoptotic response as evidenced by the increased expression of pro-apoptotic Bax and caspase-3 [114].

Different DON/ZEN co-exposure responses have been reported across a range of immune cell types. One report using porcine splenic lymphocytes concluded that DON and ZEN individually induced oxidative injury and apoptosis in a dose-dependent manner and that when combined they acted in a synergistic manner [115]. In another study in which lymphocytes derived from the *venae cava cranialis* of pigs were used, DON and ZEN were cytotoxic when used individually but behaved antagonistically (low doses) and synergistically (higher doses) with significant synergy also seen when genotoxicity was measured at 72 h [116]. In another study, mouse primary spleen T lymphocytes were used to investigate the toxic effect of ZEN and DON both alone and in combination. T cell activation by concanavalin was inhibited by both ZEN and DON, concomitant with a dose-dependent decrease in cell viability and synergistic effects were seen at 24 h following co-exposure. Immune-related functions of the activated cells were also inhibited synergistically following co-exposure [123]. Elsewhere, DON and ZEN were shown to be cytotoxic to human leukaemia monocytic THP-1 cells with evidence of an antagonistic effect at 48 hr following co-exposure [117]. Both toxins were seen to act synergistically on macrophage-derived ANA-1 cells however when cytotoxicity and the induction of apoptosis were measured. Interestingly, evidence of an inhibition of the oestrogenic effects of ZEN by DON was also reported in that study [118].

One interesting study used in vitro bi- and tri- culture models, involving human-derived Caco-2, THP-1 and HepaRG cell lines, to evaluate mycotoxin effects. No cytotoxicity was observed in the any of the bi-culture system when IC_10_ concentrations were used whereas a synergistic effect was seen using IC_30_ concentrations (see Table 4). In the tri-culture system however, no interaction was observed in response to mycotoxins in combination at IC_10_ or IC_30_. These results imply that mycotoxin efficacy was potentially being modulated by cell-cell interactions occurring during tri-culture [120]. Elsewhere, another study used a fish cell line BF-2 (in vitro) and zebrafish larvae (in vivo) to study the individual and combined effect of mycotoxins including DON and ZEN. BF-2 cells were more sensitive to DON and ZEN individually at 48 h post exposure than they were during co-exposure to both at the same timepoint, thus exhibiting an antagonistic effect. An antagonistic effect was also observed when oxidative stress-induced cell death was measured in BF-2 cells. In addition, whereas ZEN but not DON was able to induce Zebra fish embryo mortality when measured at 72 h following exposure, an antagonistic effect was observed when both mycotoxins were combined as evidenced by a decrease in embryo mortality [119]. These results from the various in vitro studies demonstrate that the toxic effect of the mycotoxins DON and ZEN can vary depending on the types of cells, concentration of the toxins and the exposure times used.

## 6. In Vivo Studies on Co-Exposure to DON and ZEN

The effects of exposure to DON and/or ZEN have also been reported following in vivo studies using rats, mice and pigs and the specific conditions used are again summarised in Table 4. One in vivo study reported on nephrotoxicity following co-exposure to DON and ZEN. Female mice were administered DON and ZEN by intraperitoneal injection and their kidneys were assessed for renal damage at 12 days post-exposure. It was found that DON and ZEN were each nephrotoxic, as measured from the levels of induced oxidative stress and renal apoptosis and that the effect of co-exposure to both was antagonistic [121]. Elsewhere, rats were used to investigate the chronic effects of mycotoxin dietary ingestion over a 14 day period. The study showed an antagonistic effect between DON and ZEN as measured in terms of absolute liver weight. Co-exposure also resulted in antagonistic effects on liver levels of glutathione and on the concentration of malondialdehyde in the kidney [122]. In a later study by the same group, the effect of lower mycotoxin concentrations and shorter treatment times (up to 5 days) was evaluated. Rats were injected with DON and/or ZEN daily for 5 days with. It was found that total glutathione levels and glutathione peroxidase activity were both increased in the livers of co-exposed animals but not in animals that were treated with DON or ZEN alone, implying a synergistic between the two mycotoxins [124]. Another study involving mice used liver and serum metabolic profiling as a means to assess DON and ZEN toxicity. The results showed that co-exposure was associated with a reduction in overall toxicity in comparison to when both toxins were used individually, again showing a clear antagonistic effect [125]. The same group later used metabolic profiling of mouse urine samples to investigate toxin co-exposure following intra-gastric administration of DON and ZEN. Once again the data showed an antagonistic response when both toxins were administered simultaneously [126]. Elsewhere, it was found that co-exposure of mice (by intraperitoneal injection) to DON and ZEN resulted in a dose-dependent and synergistic reduction in brain antioxidant activity and protein levels as well as increased apoptosis [127]. A more recent study of piglets placed on a three week controlled diet showed that neither body weight gain nor the average daily feed intake were significantly impacted following exposure to either DON or ZEN. Co-exposed piglets showed significantly lower body weight gains and average daily feed intakes however indicating that both toxins were acting synergistically to disrupt intestinal functions and caused systematic inflammation [128]. As with the in vitro studies, the in vivo studies showed that the resulting combinatorial effects of DON and ZEN can be different depending on the animals used in the study as well as the parameters measured.

## 7. Conclusions

It is clear that it is not always possible to predict the toxic effects of co-exposure to DON and ZEN by extrapolating from data obtained when the toxins are used individually. Various in vitro studies demonstrate that results can vary depending on the cell type used, the toxin concentration, and the exposure times involved. This makes it difficult to compare between the different results. The studies showed that even when the same cell type was used in different studies, the effect of DON + ZEN varied between additive, synergistic, or antagonistic. As in vitro studies can only provide information relating to specific cells, in vivo studies have also been carried out; however, similar results were obtained. It cannot be excluded that the differential co-exposure responses have been reported across a range of immune cell types, and may be due in part to variations in the cell types used and the concentrations of the toxins. Nonetheless, it is clear that across different species and cell types, for a range of concentrations, exposure times, and administration methods, it is difficult to predict and compare the combinatorial effects of the co-occurring mycotoxins DON and ZEN.

To fully answer the question of whether co-contamination of foodstuffs with both DON and ZEN imparts a synergistic or an antagonistic effect, it is clear that further research is required to understand the interactions of the combined mycotoxins. Only then can we fully start to appreciate the potential scale of any combinatorial impact when foodstuffs contaminated with both mycotoxins are ingested, and effectively develop strategies to successfully mitigate against the effects caused.

## Figures and Tables

**Table 1:** Mycotoxins, fungal species that produce them, and their reported health effects [10,11,12,13,14].

Mycotoxin	Fungal Species	Human Health Effects
Aflatoxins	*Aspergillus*	Haemorrhage, liver damage. carcinogenesis, gastrointestinal dysfunction, anaemia, jaundice, reduced reproductivity
Trichothecenes	*Fusarium*	Growth stunt, reproductive disorder, vomiting, feed refusal, reduced ovarian function
Ochratoxins	*Aspergillus, Penicillium*	Carcinogenesis, nephrotoxicity
Zearalenone	*Fusarium*	Hormonal imbalance, oestrogenic effects
Fumonisins	*Fusarium*	Nephrotoxicity, esophageal cancer

**Table 2:** Maximum EFSA levels of DON in various foodstuffs. Table adapted from Commission Regulation (EC) No. 1881/2006 of 19 December 2006 (Section 2) and Commission Regulation (EC) 17 August 2006 on the presence of deoxynivalenol, zearalenone, ochratoxin A, T-2 and HT-2 and fumonisins in products intended for animal feeding [37,38].

**Foodstuff**	**Maximum Level (µg/kg)**
Unprocessed cereals except durum wheat, oats and maize	1250
Unprocessed durum wheat and oats	1750
Unprocessed maize except for unprocessed maize intended to be processed by wet milling	1750
Cereals intended for direct human consumption, cereal flour, bran and germ as end product marketed for direct human consumption	750
Dry pasta	750
Bread, pastries, biscuits, cereal snacks and breakfast cereal	500
Processed cereal based foods and baby foods for infants and young children	200
**Products Intended for Animal Feed**	**Guidance Value Relative to a Feedingstuff with a Moisture Content of 12% (µg/kg)**
Cereals and cereal products with the exception of maize by-products	8000
Maize by-products	12,000
Complementary and complete feedingstuff (with the exception of those listed below)	5000
Complementary and complete feedingstuff for pigs	900
Complementary and complete feedingstuff for calves (<4 months), lambs, and kids	2000

**Table 3:** Maximum EFSA levels of ZEN in various foodstuffs. Table from Commission Regulation (EC) No. 1881/2006 of 19 December 2006 (Section 2) and Commission Regulation (EC) 17 August 2006 on the presence of deoxynivalenol, zearalenone, ochratoxin A, T-2 and HT-2 and fumonisins in products intended for animal feeding [37,38].

**Foodstuff**	**Maximum Level (µg/kg)**
Unprocessed cereals other than maize	100
Unprocessed maize except for unprocessed maize intended to be processed by wet milling	350
Cereals intended for direct human consumption, cereal flour, bran, and germ as the end product marketed for direct human consumption	75
Bread (including small bakery wares), pastries, biscuits, cereal snacks, and breakfast cereals, excluding maize-based snacks and maize-based breakfast cereals	50
Maize intended for direct human consumption, maize-based snacks and maize-based breakfast cereals	100
Processed cereal based foods (excluding processed maize-based foods) and baby foods for infants and young children	20
Processed maize-based foods for infants and young children	20
**Products Intended for Animal Feed**	**Guidance Value Relative to a Feedingstuff with a Moisture Content of 12% (µg/kg)**
Cereals and cereal products with the exception of maize by-products	2000
Maize by-products	3000
Complementary and complete feedingstuff for piglets and gilts (young sows)	100
Complementary and complete feedingstuff for sows and fattening pigs	250
Complementary and complete feedingstuff for calves, dairy cattle, sheep (including lamb) and goats (including kids)	500

## Data Availability

Not applicable.
